# Comparing different sperm concentrations for optimizing cooled semen use in the dog

**DOI:** 10.3389/fvets.2023.1339840

**Published:** 2024-01-29

**Authors:** Nicole Sugai, Stephen Werre, Julie T. Cecere, Orsolya Balogh

**Affiliations:** ^1^Department of Small Animal Clinical Sciences, Virginia-Maryland College of Veterinary Medicine, Blacksburg, VA, United States; ^2^Department of Population Health Sciences, Virginia-Maryland College of Veterinary Medicine, Blacksburg, VA, United States

**Keywords:** canine, concentration, chilled, sperm, viability, morphology

## Abstract

The use of shipping canine semen for artificial insemination has bloomed over the last 20 years. This allows for the spread of genetic material while overcoming geographical or time-related challenges. The optimal sperm concentration for cooled semen transport in the dog is unknown. Often canine semen is extended 1:3–5 vol:vol without standardized sperm concentrations for cooled shipment. We compared different sperm concentrations for cooled storage and hypothesized that lower concentrations would result in better semen quality. Semen was collected from healthy client-owned dogs (*n* = 8). Individual ejaculates were divided into a control aliquot (CON) extended 1:3 vol:vol with a commercial extender. The remaining sample was centrifuged and extended to 200 ×10^6^ sperm/ml (C200), then serially diluted to 100, 50, and 25 ×10^6^ sperm/ml concentrations (C100-C25). Aliquots were cooled for 24 h and then centrifuged and re-extended. Sperm concentration, plasma membrane integrity (PMI, %), motility (subjective total, STM; computer-assisted sperm analysis (CASA) total and progressive, TM, PM; %), and normal morphology (NM, %) were assessed in raw semen (T0), post-extension (T1), after 24 h of cooling (T2), and after processing at 24 h (T3). Cooling resulted in significant declines in STM and NM for all groups and in decreased PMI for CON and C25-50. After cooling (at T2), PMI was significantly lower for C25 compared with all the groups and higher for CON compared with C25-100 (*p* ≤ 0.038). Processing and re-extension after cooling further decreased the spermiogram parameters. At T3, PMI for CON was similar to C200 but significantly higher than C25-100, while C25 had the lowest PMI. For motility parameters and NM, C25 performed worse than all or most of the other groups. Comparing CON at T3 with C25-200 at T2, PMI, STM, and NM for CON were significantly lower than C25-200, C200, and C100-200, respectively. In conclusion, our results show that cooling canine semen for 24 h at 200 ×10^6^ sperm/ml final concentration after processing or extending 1:3 vol:vol without centrifugation is preferred based on the highest PMI. If volume restrictions apply, processing raw semen and extending to the desired volume with higher sperm concentrations at the collection facility is superior to centrifugation and volume adjustment after 24 h of cooled storage.

## 1 Introduction

There has been a significant increase in the use of shipped cooled semen for artificial insemination (AI) in dogs (1, 2). This is due to reduced stress on animal movement and easier logistics for short-term sperm storage. The use of cooled semen for breeding management is superior to frozen semen as reflected in improved pregnancy rates, allowing for the dissemination of canine genetics across the globe (2, 3). Certain drawbacks such as quality assurances and lack of standardization of processing procedures can hinder the success rates of cooled semen (4). This can lead to a significant decline in semen quality at the time of insemination, resulting in decreased pregnancy rates and litter sizes (3, 5).

Similar to equine semen, shipped canine semen is typically received for AI purposes 24 to 48 h after initial collection. Fertility of cooled semen is not maintained for longer than 24 to 48 h in a cooled, *in vitro* state. This is reflected in a reduction in biologically acceptable outcomes, such as pregnancy rates, when cooled semen is inseminated after 48 h in storage (1, 6, 7). Discrepancies in pregnancy rates and spermiogram parameters from semen stored up to 7–10 days with *in vivo* or *in vitro* conditions have been studied and previously reported for stallions and dogs. (2, 8–10). The time spent in cooled storage leads to a decline in semen quality by increasing reactive oxygen species (ROS) and the accumulation of moribund sperm. Ultimately, this combination negatively impacts fertility and pregnancy rates (7, 9, 11–13).

Cooling is a significant stressor on sperm cells, and considerable work in canine andrology needs to be performed to establish appropriate protocols for shipping. Specifically, previous studies have addressed extender formulations, packaging, centrifugation parameters, and cooling rates (14–17). Processing the ejaculate before shipping removes the seminal plasma and prostatic fluid, which results in improved post-cooling sperm motility and quality parameters (11, 18, 19). For stallions and dogs, reducing the amount of seminal plasma to 10–25% of the initial volume of the ejaculate was shown to balance its beneficial effects on spermatozoa longevity (20–22). The complete removal of seminal plasma eliminates its protective and beneficial elements during sperm storage and transport in the female reproductive tract (21, 23, 24). Centrifugation parameters for canine semen have also been established with a range between 400 and 900 X g for 5–10 min, resulting in minimal sperm losses and acceptable quality (14, 17).

The addition of appropriately formulated extenders to the semen reduces the negative metabolic impact and permits adequate metabolic activity during cooled storage (13, 24). The amount of extender used can negatively impact sperm viability if the semen is excessively diluted, causing osmolarity changes during storage that impact spermatozoa function (5, 8, 24). Therefore, finding the appropriate sperm concentration and/or dilution rate is of utmost importance for successful canine semen cooling. In equids, cooled-shipped semen is routinely used and processed based on the standards established over the last 30 years. For stallions, sperm concentrations of 100 ×10^6^ sperm/ml showed drastic declines in semen quality parameters over time in 25°C storage when compared with 25 to 50 × 10^6^ sperm/ml concentrations (5, 24). To date, there is no study in dogs directly comparing the spermiogram after cooled semen storage with differing sperm concentrations. Previous studies on different aspects of cooled canine semen (e.g., different cooling rates or extenders) used final extended sperm concentrations from 25 to 300 × 10^6^ sperm/mL or a semen:extender ratio of 1:3 or 1:5 vol:vol dilution (6, 8, 11, 14–16). However, these results are not directly comparable and cannot be used as the basis to determine optimal sperm concentrations because the experimental conditions were different between studies and used pooled semen samples. In contrast, for cryopreserved canine sperm, optimal concentrations between 100 and 200 × 10^6^ sperm/ml have been established and are routinely used in the industry (25–27).

The ideal sperm concentration allows for minimal reduction in sperm quality after cooled storage. *In vitro* parameters frequently used for estimating fertility include sperm plasma membrane integrity (PMI), motility parameters, and percentage normal morphology (3, 28). The objective of our study was to investigate the effects of different sperm concentrations, i.e., between 25 and 200 × 10^6^ sperm/ml, on semen quality by assessing the previously mentioned *in vitro* parameters during 24 h of cooled storage. Our goal was to establish an optimal concentration range for shipped cooled semen that can be recommended for routine use in the clinical setting. Our hypothesis was that lower concentrations of 25 and 50 × 10^6^ sperm/ml would result in better sperm quality. Furthermore, we evaluated the effect of re-centrifugation after cooled storage to mimic clinical scenarios where volume adjustments are needed prior to insemination. In contrast to most canine studies, we used individual samples to control for individual dog effects and prevent interference or bias from cross reaction between dogs in pooled semen samples, which may result in poor spermiogram outcomes.

## 2 Materials and methods

### 2.1 Animals

Eight healthy, adult dogs of medium to large breeds (mean 32.7 kg, range 24.8 to 39.4 kg) and between 2 and 7 years of age (mean 4.3 years, range 2.5 to 6.5 years) were included in this study. The dogs were of the following breeds: German Shepherd (*n* = 2), Golden Retriever (*n* = 3), Labrador Retriever (*n* = 2), and Weimaraner (*n* = 1). At the time of enrollment and semen collection, all participants were found to be in good general and reproductive health and not currently on any medications that could interfere with sperm quality. *Brucella canis* serology (*Brucella Canis* Multiplex, Cornell University Animal Health Diagnostic Center) was negative for all dogs at the time of inclusion. Six dogs were proven studs and two dogs were maiden/unproven with no family history of infertility. All dogs were client-owned and enrolled on a voluntary basis in compliance with Virginia-Maryland College of Veterinary Medicine Institutional Animal Care and Use Committee (IACUC protocol number 21–194) after owners signed an informed consent form.

### 2.2 Semen collection

Dogs were allowed for a minimum of 7 to 10 days of sexual rest before enrolling in the study. Semen collection was performed by digital stimulation as previously described (29). The first and second fractions were collected into disposable plastic funnel sleeves (Minitube, Tiefenbach, Germany), transferred to 15 mL conical Falcon tubes (Corning, Christiansburg, VA, United States), and kept at room temperature during initial processing. Only ejaculates with at least 200 ×10^6^ total sperm and 0.5 mL total volume, a minimum of 100 ×10^6^ sperm/mL concentration, ≥70% subjective total motility, and ≥ 40% morphologically normal spermatozoa were included in the study.

### 2.3 Initial semen evaluation

Initial evaluation of the fresh (raw) ejaculate was performed immediately after collection in the same manner as previously described (17). In brief, spermatozoa concentration and PMI were determined using the Nucleocounter^®^ SP-100™ with SP1 cassettes, according to the manufacturer’s instructions (Chemometec, Allerød, Denmark). The total number of spermatozoa in the ejaculate was calculated as concentration multiplied by the total ejaculate volume. PMI (%) was calculated as follows: (total sperm concentration – concentration of non-viable sperm)/(total sperm concentration) x100. Subjective total motility (STM, %) was assessed by placing 10 μL of a well-mixed, undiluted semen sample on a pre-warmed (37°C) glass slide under a coverslip and examined using a phase-contrast microscope at 100X magnification. Computer-assisted sperm analysis (CASA) for total motility (TM, %) and progressive motility (PM, %) was performed using Sperm Vision™ (Minitube, Tiefenbach, Germany) as previously described (17). Semen samples were diluted with warmed phosphate buffered saline if needed to achieve 25–50 ×10^6^ sperm/ml concentrations for all CASA evaluations. Samples were assessed using pre-warmed Leija 20 µm disposable counting chamber slides (Minitube, Tiefenbach, Germany). All motility evaluations were run at 37°C using the settings presented in Table 1. For morphology evaluations, an eosin–nigrosin slide was made using 10 μL of well-mixed semen sample and 10 μL of eosin–nigrosin stain (Hancock stain, Animal Reproduction Systems Inc., Ontario, CA, United States), which were mixed to make a monolayer. Sperm morphology was read by the same two evaluators blinded to each other and categorized into normal morphology (NM, %) and abnormalities (%) related to acrosome, head, midpiece, and tail defects.

**Table 1:** Technical settings for the computer-assisted sperm analysis (CASA) system (Sperm Vision™, Minitube).

Parameter	Setting
Field-of-view depth (sample chamber depth)	20 μm
Light adjustment	80–110
Total number of fields	7 fields
Sperm recognition area	20–60 μm^2^
Frame rate	60 frames/s
Points assessed for sperm motility	11
Total motility	Progressive motility + local motility
Immotile sperm	AOC < 9.5
Local motility	DSL < 6.0 μm
Progressive motility	Every cell that is not “immotile” or “local motile”
Hyperactive sperm	VCL > 118 μm/s, ALH > 6.5 μm and LIN < 0.5
Linear sperm	STR > 0.9 and LIN > 0.5
Non-linear sperm	STR ≤ 0.9 and LIN ≤ 0.5
Curvilinear sperm	DAP/Radius ≥ 3 and LIN < 0.5

### 2.4 Semen processing and extension

After initial semen evaluation, an aliquot of the raw semen was placed in a separate 15 mL Falcon tube and extended to 1:3 vol:vol ratio with warmed (37°C) CaniPlus Chill LT (Minitube, Tiefenbach, Germany), which served as the control treatment (CON). The final sperm concentration in the CON samples was 23.37–182.10×10^6^ sperm/mL (mean ± SD, 54.99 ± 49.81 ×10^6^ sperm/mL), and this was maintained throughout the study. The remaining ejaculate was placed in a 15 mL conical Falcon tube and centrifuged at room temperature at 720 *g* for 10 min (17) in a Sorvall ST8R centrifuge (Thermo Scientific, Waltham, MA, United States) with a swinging bucket rotor and soft acceleration/deceleration. After centrifugation, the supernatant was immediately removed from the sperm pellet leaving 10 to 20% of initial seminal plasma volume. Afterwards, the pellet was extended with CaniPlus Chill LT to yield 200 ×10^6^ sperm/mL concentration (C200), followed by serial dilutions (1:1 vol:vol extension for each subsequent concentration) to achieve 100, 50, and 25 ×10^6^ sperm/mL (C100, C50, and C25 treatment groups). Each aliquot was placed in a 15 mL Falcon tube for the remainder of the study. Immediately after extension, PMI, motility, and morphology were determined for each group as described above. This evaluation time point was recorded as T1.

### 2.5 Cooling, re-centrifugation, and re-extension

The extended semen aliquots were packaged in a canine semen transport box (Minitube, Tiefenbach, Germany) to mimic clinical shipping conditions as previously described (17). The cooling process was passive for the samples to be kept at approximately 4°C for 24 h. After 24 h of cooled storage, spermatozoa PMI, motility, and morphology were determined, and this time point was recorded as T2. After these evaluations were finished, all aliquots were re-processed as follows: centrifuged at 720 *g* for 10 min, the supernatant was removed, and the pellet was re-extended with CaniPlus Chill LT to the initial pre-centrifugation volume. The re-extended aliquots were evaluated again for PMI, motility, and morphology parameters, and this time point was recorded as T3.

**Figure 1:**
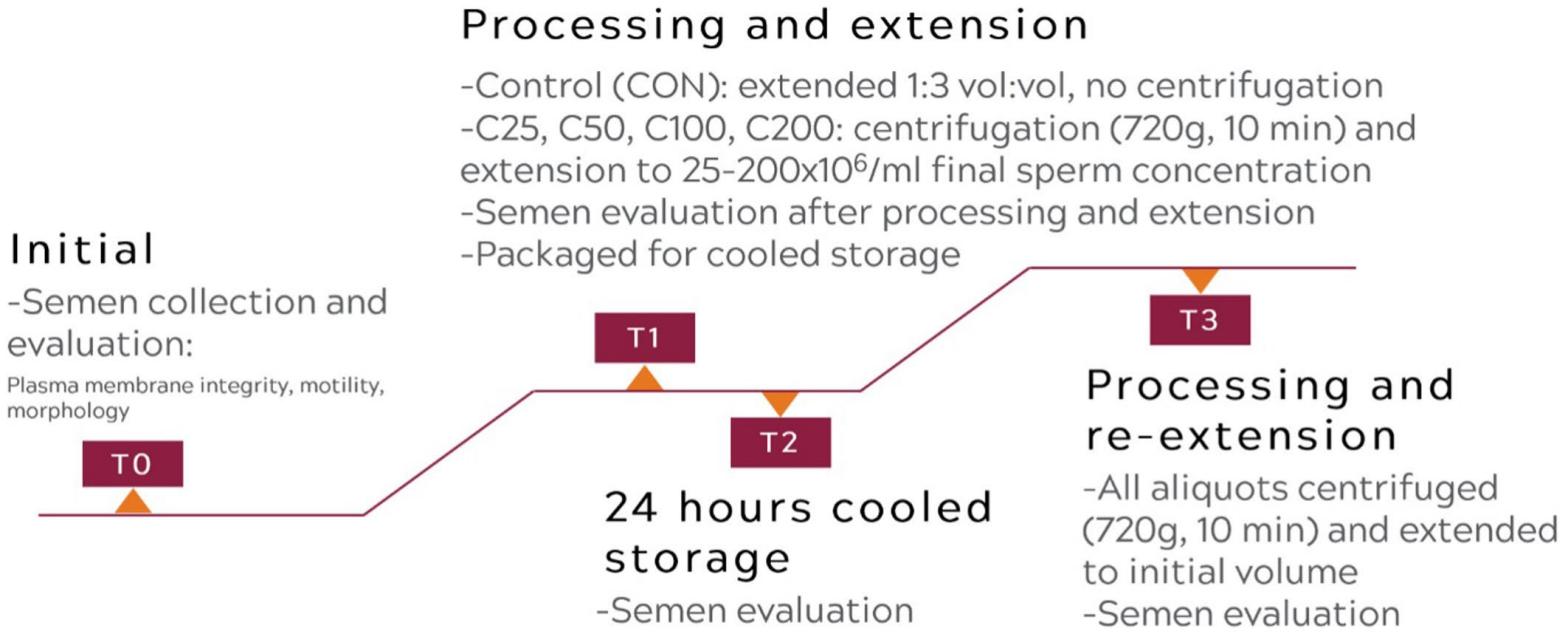
Flowchart of the experimental procedure.

A flowchart describing the experimental design is presented in Figure 1.

### 2.6 Statistical analysis

Normal probability plots were inspected to assess the normal distribution of data. A mixed-model analysis of covariance (ANCOVA) was used to assess the effects of time and treatment on semen parameters (PMI, STM, CASA TM and PM, NM, and morphological defects). The initial raw (T0) semen evaluation parameters were included as covariates in the model to adjust for differences in individual dog ejaculate characteristics. The model specified treatment (groups CON, C25–C200), time (T1: after initial processing and extension, T2: after 24 h of cooling, and T3: after processing and re-extension following 24 h of cooling), and the interaction between treatment and time as fixed effects. Dog identification was included in the model as the subject of repetition. Repetition within the subject was modeled using the compound symmetry matrix specification. The interaction term was further analyzed to compare treatments within each time point and time points within each treatment. *p*-values were adjusted for multiple comparisons using the Tukey–Kramer test.

Additionally, we also compared the CON group at time T3 with all other groups (C25–C200) at time T2. This was conducted to assess the performance of CON, where the initial extension was performed without centrifugation, but the sample may need re-processing after 24 h of cooling for volume adjustment. For this analysis, a mixed-model ANCOVA was used to assess treatment effects (CON at T3, C25–C200 at T2) with T0 used as a covariate, adjusting for multiple comparisons using the Dunnett–Hsu test. Dog identification was included in the model as the subject of repetition. Repetition within the subject was modeled using the compound symmetry matrix specification.

All statistical analyses were performed with procedures available in SAS version 9.4 (Cary, NC, United States). Values were considered statistically significant when *p* < 0.05. PMI, STM, TM, PM, and sperm morphology data are shown as mean ± standard error (SEM) unless otherwise stated.

## 3 Results

### 3.1 Effect of sperm concentration and cooling on plasma membrane integrity

**Figure 2:**
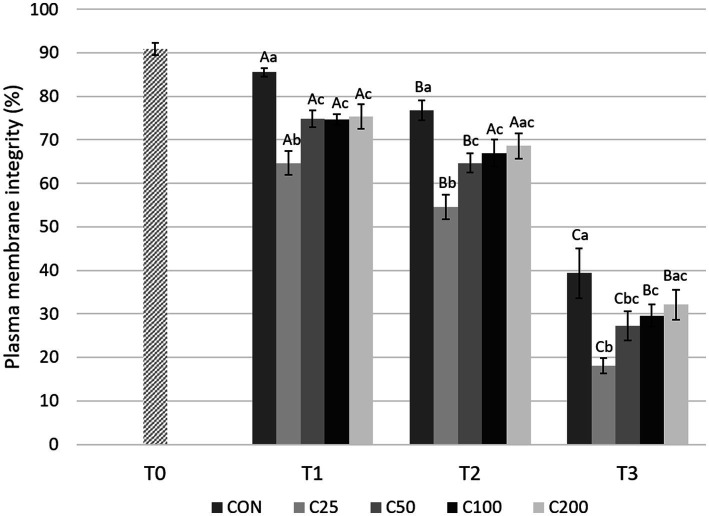
Spermatozoa plasma membrane integrity for each group at each time point from T0 to T3. Grayscale-colored bars denote the mean and whiskers the standard error of the mean (SEM) per treatment group at each time point. Treatment groups labeled as CON: control, C25: 25 × 10^6^ sperm/mL, C50: 50 × 10^6^ sperm/mL, C100: 100 × 10^6^ sperm/mL, and C200: 200 × 10^6^ sperm/mL sperm concentration. Time points are T0: initial, raw semen evaluation, T1: fresh semen after processing and extension, T2: after 24 h of cooling, and T3: semen processed and re-extended after 24 h of cooled storage. Different capital letters (A, B, and C) denote statistically significant differences between time points for each group. Different lowercase letters (a, b, and c) denote statistically significant differences between groups at specific time points.

There was a significant decline in PMI over time for all groups (Figure 2). An initial significant decrease in PMI during cooling from T1 to T2 was noted for groups CON, C25, and C50 (*p* ≤ 0.034) but not for C100 and C200. Afterwards, PMI declined significantly from T2 to T3 in all groups (*p* < 0.0001) in response to post-cooling processing at 24 h.

**Figure 3:**
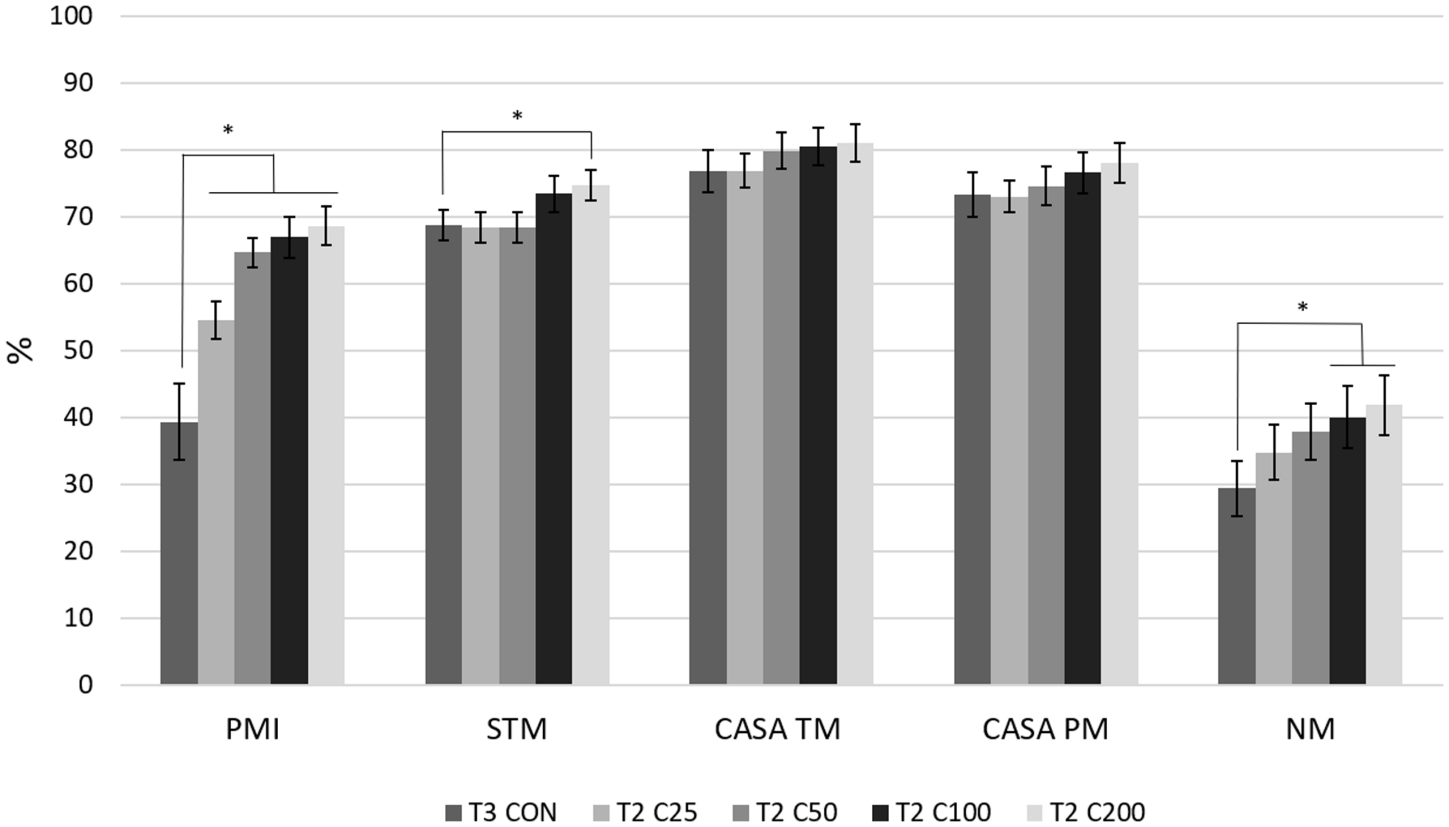
Comparison of spermiogram parameters between the control group after processing and re-extension at 24 h (labeled T3 CON) and the groups C25–C200 after 24 h of cooled storage (labeled T2 C25 through T2 C200). C25: 25 million/mL, C50: 50 million/mL, C100: 100million/mL, and C200: 200 million/mL sperm concentration. The grayscale-colored bars denote the mean and whiskers the standard error of the mean (SEM). PMI: plasma membrane integrity, STM: subjective total motility, CASA TM and PM: total and progressive motility by computer-assisted sperm analysis, NM: normal morphology. Asterisks denote statistically significant differences between the groups.

Comparing differences between the groups at each time point, PMI for CON was higher compared with all other treatment groups (C25–C200) at T1 (*p* ≤ 0.030) and compared with C25–C100 at T2 and T3 (*p* ≤ 0.038 and *p* ≤ 0.043, respectively). PMI for CON did not differ from C200 at times T2 and T3 (*p* ≥ 0.126). PMI for C25 was significantly lower than all other groups at T1 and T2 (*p* ≤ 0.036) and compared with groups CON, C100, and C200 at T3 (*p* ≤ 0.009). Groups of C50–C200 were not significantly different from each other at any time point (Figure 2). Comparison of CON at T3 to the other treatment groups at T2 showed significantly lower PMI for CON than for C25–C200 (*p* ≤ 0.001, Figure 3).

### 3.2 Effect of sperm concentration and cooling on motility

**Figure 4:**
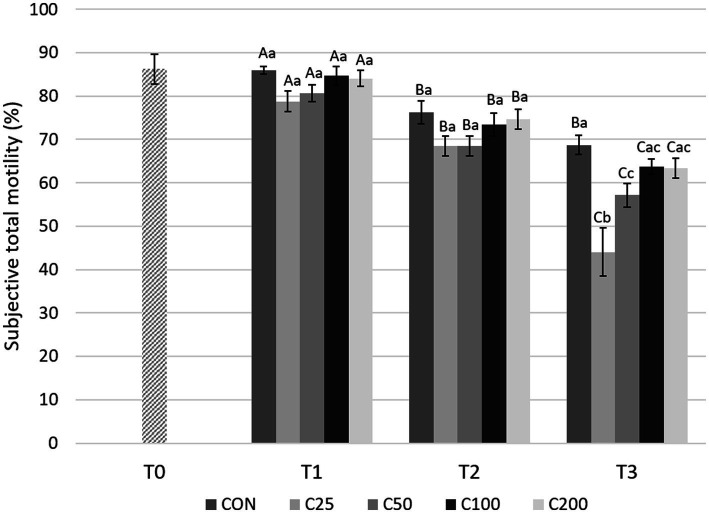
Subjective total motility for each group at each time point from T0 to T3. Grayscale-colored bars denote the mean and whiskers the standard error of the mean (SEM) per treatment group at each time point. Treatment groups labeled as CON: control, C25: 25 × 10^6^ sperm/mL, C50: 50 × 10^6^ sperm/mL, C100: 100 × 10^6^ sperm/mL, and C200: 200 × 10^6^ sperm/mL sperm concentration. Time points are T0: initial, raw semen evaluation, T1: fresh semen after processing and extension, T2: after 24 h of cooling, and T3: semen processed and re-extended after 24 h of cooled storage. Different capital letters (A, B, and C) denote statistically significant differences between time points for each group. Different lowercase letters (a, b, and c) denote statistically significant differences between groups at specific time points.

There was a gradual, significant decline in STM over time (from T1 to T2 and T2 to T3) for all groups (*p* ≤ 0.013), except for CON from T2 to T3 (Figure 4). Comparisons between the groups showed no significant differences in STM at T1 and T2 (*p* ≥ 0.18 and *p* ≥ 0.12, respectively). After processing at 24 h (T3), STM was higher for CON compared with C25 and C50 (*p* ≤ 0.005), and C25 was also lower than C50–C200 (*p* ≤ 0.0009).

**Figure 5:**
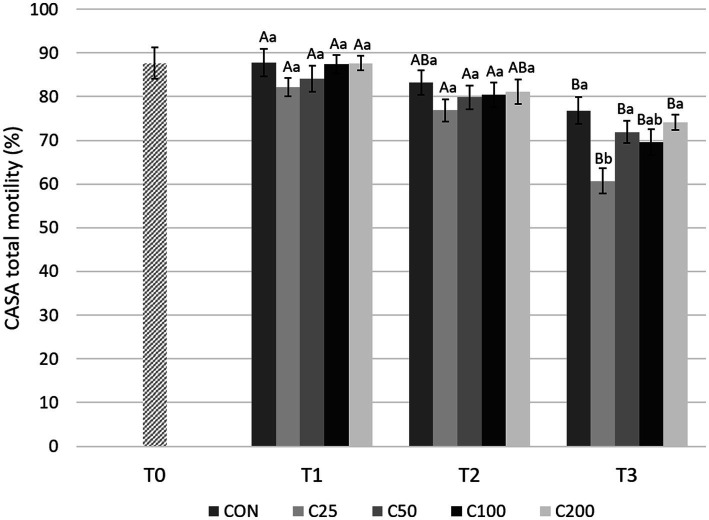
CASA total motility for each group at each time point from T0 to T3. Grayscale-colored bars denote the mean and whiskers the standard error of the mean (SEM) per treatment group at each time point. Treatment groups labeled as CON: control, C25: 25 × 10^6^ sperm/mL, C50: 50 × 10^6^ sperm/mL, C100: 100 × 10^6^ sperm/mL, and C200: 200 × 10^6^ sperm/mL sperm concentration. Time points are T0: initial, raw semen evaluation, T1: fresh semen after processing and extension, T2: after 24 h of cooling, and T3: semen processed and re-extended after 24 h of cooled storage. Different capital letters (A, B, and C) denote statistically significant differences between time points for each group. Different lowercase letters (a, b, and c) denote statistically significant differences between groups at specific time points.

CASA TM decreased significantly from T1 to T3 for all groups (*p* ≤ 0.004), but there were no significant changes between T1 and T2 during cooling (Figure 5). A significant decline in TM was noted from T2 to T3 for the groups C25–C100 (*p* ≤ 0.049). CASA TM was similar between the groups at T1 and T2 (*p* ≥ 0.320). At time T3, TM for C25 was lower than CON (*p* < 0.0001), C50, and C200 (*p* ≤ 0.01) (Figure 5).

**Figure 6:**
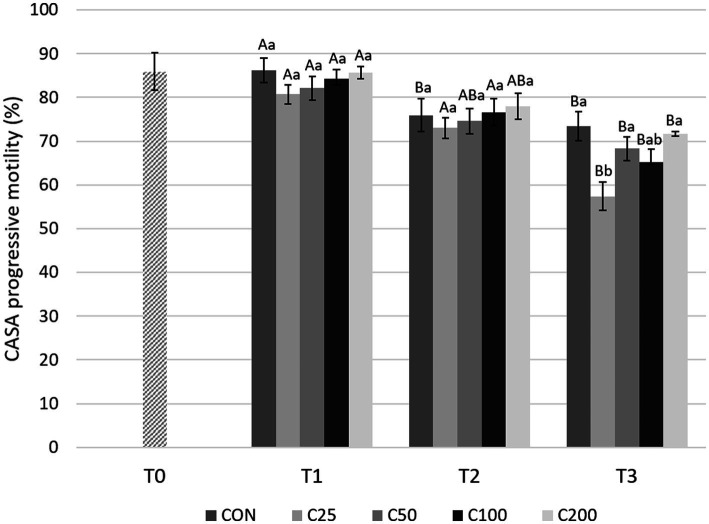
CASA progressive motility for each group at each time point from T0 to T3. Grayscale-colored bars denote the mean and whiskers the standard error of the mean (SEM) per treatment group at each time point. Treatment groups labeled as CON: control, C25: 25 × 10^6^ sperm/mL, C50: 50 × 10^6^ sperm/mL, C100: 100 × 10^6^ sperm/mL, and C200: 200 × 10^6^ sperm/mL sperm concentration. Time points are T0: initial, raw semen evaluation, T1: fresh semen after processing and extension, T2: after 24 h of cooling, and T3: semen processed and re-extended after 24 h of cooled storage. Different capital letters (A, B, and C) denote statistically significant differences between time points for each group. Different lowercase letters (a, b, and c) denote statistically significant differences between groups at specific time points.

With regard to CASA PM evaluations (Figure 6), there was a significant decline from T1 to T3 for all the groups (*p* ≤ 0.001). A decrease in PM from T1 to T2 was found only in the CON group (*p* = 0.013) and from T2 to T3 in groups C25 and C100 (*p* ≤ 0.005). PM was similar between the groups at T1 and T2 (*p* ≥ 0.531 and *p* ≥ 0.623, respectively). At T3, PM for C25 was significantly lower compared with CON, C50, and C200 (*p* ≤ 0.022) (Figure 6).

Comparing motility parameters between CON at T3 and C25–C200 at T2 (Figure 3), STM differed only between C200 and CON (*p* = 0.039). CASA TM and PM did not differ between the groups (*p* ≥ 0.339).

### 3.3 Effect of sperm concentration and cooling on morphology

**Figure 7:**
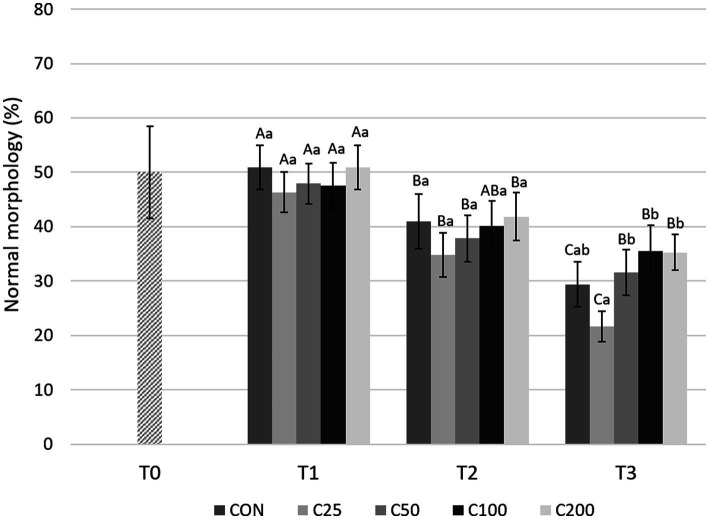
Normal morphology for each group at each time point from T0 to T3. Grayscale-colored bars denote the mean and whiskers the standard error of the mean (SEM) per treatment group at each time point. Treatment groups labeled as CON: control, C25: 25 × 10^6^ sperm/mL, C50: 50 × 10^6^ sperm/mL, C100: 100 × 10^6^ sperm/ml, and C200: 200 × 10^6^ sperm/mL sperm concentration. Time points are T0: initial, raw semen evaluation, T1: fresh semen after processing and extension, T2: after 24 h of cooling, and T3: semen processed and re-extended after 24 h of cooled storage. Different capital letters (A, B, and C) denote statistically significant differences between time points for each group. Different lowercase letters (a, b, and c) denote statistically significant differences between groups at specific time points.

Morphologic abnormalities were grouped by region of the spermatozoa in which they occurred, i.e., acrosome, head, midpiece, and tail abnormalities. As shown in Figure 7, NM decreased significantly from T1 to T2 during cooling in all groups (*p* ≤ 0.015) except C100 (*p* = 0.054), and from T2 to T3 in CON and C25 (*p* ≤ 0.001). The decline from T1 to T3 was substantial for all the groups (*p* ≤ 0.0008). Looking at each time point, NM was similar across treatments at T1 and T2 (*p* ≥ 0.612 and *p* ≥ 0.189, respectively). At time T3, NM of C25 was lower than C50–C200 (*p* ≤ 0.020; Figure 7).

There were significant changes in certain sperm abnormalities over time and across groups (please see Table 2). Acrosome defects increased in all groups during cooling (from T1 to T2, *p* ≤ 0.009) and after processing and re-extension (from T2 to T3) in groups C25 and C50 (*p* ≤ 0.05). There were no group-specific differences at T1 and T2 (*p* ≥ 0.375), but at time T3, acrosome abnormalities were most prevalent in C25 and significantly higher than in all other groups (*p* ≤ 0.002). Additionally, acrosome defects at T3 were higher in C50 than in C200 (*p* = 0.034). There were no changes in sperm head abnormalities over time except for a decline in CON, C25, and C50 by T3 (*p* ≤ 0.011), and the groups were not different at any time point (*p* ≥ 0.263). The percentage of midpiece defects remained unchanged during cooling (T1 to T2) in all groups (*p* ≥ 0.115) but increased from T1 to T3 in CON, C25, and C200 (*p* ≤ 0.023) and from T2 to T3 in CON (*p* = 0.009). The groups were not different in terms of midpiece defects at any time point (p ≥ 0.375). There were no time- or treatment-related differences in tail abnormalities (*p* ≥ 0.140).

**Table 2:** Changes in specific sperm morphological defects over time and per treatment group.

Morphological defects by sperm region	Groups	T0	T1	T2	T3
Acrosome	CON	4.75 ± 9.82	8.6 ± 3.3^A^	16.7 ± 2.7^B^	22.4 ± 3.4^Bac^
	C25		13.1 ± 1.6 ^A^	19.7 ± 1.9^B^	33.0 ± 1.7^Cb^
	C50		9.5 ± 0.91^A^	17.2 ± 1.7^B^	23.3 ± 2.7^Ca^
	C100		8.6 ± 1.6^A^	18.4 ± 2.7^B^	19.9 ± 2.7^Bac^
	C200		8.4 ± 1.4^A^	17.0 ± 2.5^B^	15.9 ± 1.5^Bc^
Head	CON	9.5 ± 4.17	18.9 ± 4.0^A^	17.8 ± 3.3^A^	12.2 ± 2.9^B^
	C25		17.0 ± 4.1^A^	14.7 ± 3.4^AB^	11.6 ± 2.9^B^
	C50		18.3 ± 4.1^A^	16.7 ± 2.8^AB^	12.9 ± 2.9^B^
	C100		17.3 ± 3.3^A^	16.5 ± 3.7^A^	14.1 ± 3.6^A^
	C200		18.6 ± 2.5^A^	15.9 ± 3.2^A^	15.3 ± 3.4^A^
Midpiece	CON	12.0 ± 4.87	21.7 ± 2.4^A^	22.5 ± 3.7^A^	33.7 ± 5.1^B^
	C25		21.2 ± 3.2^A^	28.7 ± 3.7^AB^	31.2 ± 2.6^B^
	C50		22.4 ± 3.6^A^	26.9 ± 2.7^A^	30.3 ± 3.2^A^
	C100		22.9 ± 1.8^A^	22.9 ± 2.4^A^	28.5 ± 3.3^A^
	C200		18.0 ± 2.5^A^	22.0 ± 2.4^AB^	30.2 ± 3.1^B^
Tail	CON	8.5 ± 2.74	2.9 ± 1.4	2.2 ± 0.67	2.3 ± 0.80
	C25		2.3 ± 0.53	2.2 ± 0.69	2.6 ± 0.76
	C50		1.8 ± 0.65	1.4 ± 0.48	1.7 ± 0.53
	C100		3.1 ± 1.3	2.0 ± 0.79	2.0 ± 0.64
	C200		3.7 ± 1.5	2.9 ± 0.73	2.7 ± 1.1

The results are presented as mean (%) and standard error of the mean (SEM, %). Groups labeled as CON: control, C25: 25 × 10^6^ sperm/mL, C50: 50 × 10^6^ sperm/mL, C100: 100 × 10^6^ sperm/mL, and C200: 200 × 10^6^ sperm/mL. Time points are T0: initial, raw semen evaluation, T1: fresh semen after processing and extension, T2: after 24 h of cooling, and T3: semen processed and re-extended after 24 h of cooled storage. Different capital letters (A, B, and C) denote significant differences between time points for each group. Different lowercase letters (a, b, and c) denote statistically significant differences between the groups at specific time points. No superscript letters denote no statistical difference.

When morphology was compared between CON at T3 and C25-200 at T2, C100 and C200 had higher NM than CON (*p* ≤ 0.011; Figure 3). Acrosome and tail abnormalities were not significantly different, while CON had lower percentages of head defects than C50 and C100 (*p* ≤ 0.038) and higher percentages of midpiece abnormalities than C200 (*p* = 0.043).

## 4 Discussion

In this study, we compared canine semen samples extended to different sperm concentrations and stored cooled for 24 h. Cooling negatively affected the spermiogram over time (from T1 to T2); the changes were dependent on the sperm parameter, and the magnitude of changes was affected by treatment, i.e., sperm concentration. For example, NM decreased significantly in all groups as we consider the decline in the C100 group biologically relevant (*p* = 0.054), with an increasing number of defects primarily affecting the acrosome. A significant decline in PMI was noted only in the CON, C25, and C50 groups and not at higher sperm concentrations. STM decreased significantly during cooling in all the groups, while CASA TM did not change and PM declined only in CON. Our findings were expected and aligned with previous studies that show a significant decline in canine sperm quality parameters in response to cooling stressors over time (2, 14, 30). These anticipated declines are based on the understanding that cooling induces ROS production with subsequent detrimental effects on the sperm (11).

Previous studies across species showed that progressive motility and acrosome integrity change over time with cold storage and can be indicators of fertility concerns (13, 14, 18). These changes follow ROS generation and induction of *in vitro* capacitation during cooled storage (31, 32). Like our findings, an increase in acrosomal abnormalities was observed previously in cooled and cryopreserved canine semen (19, 30, 31, 33–35). The changes in acrosome integrity are due to glycoprotein and glycolipid shifts and reorganization of the phospholipid bilayer, which occurs when extenders or seminal plasma proteins are not able to prevent capacitation during cold storage (32, 36, 37). Other morphologic regions of the sperm were not affected during cooling and would be less likely biologically significant to affect the fertilizing ability of the sperm (Table 2).

With respect to group comparisons, there were significant treatment-related differences at all time points. At T1, the significantly higher PMI of CON compared with the other treatment groups (mean, 85.5% versus 64.7 to 75.4%) is likely related to the negative effects of centrifugation imposed on the C25–C200 groups. This is consistent with our previous study showing an 8.65–9.78% decrease in PMI in response to centrifugation at 400–900 X g for 5–10 min (17) and is similar to findings in other studies for dog and stallion semen when assessing sperm viability parameters (14, 38, 39). During the cooling period from T1 to T2, motility (STM, CASA TM, and PM) and morphology parameters were not affected by the different sperm concentrations. In contrast, after 24 h of cooling (T2), PMI for CON was significantly higher compared with C25–C100 but similar to C200. C25 performed worst in terms of PMI, which was significantly lower than in all other groups both at T1 and T2. These findings indicate that extension of the fresh canine ejaculate at 1:3 vol:vol without centrifugation (CON, final concentration of 23.37–182.10 x10^6^sperm/mL) or to 200 × 10^6^ sperm/mL concentration after centrifugation with removal of the majority of the seminal plasma produces superior results after 24 h of cooling. Centrifugation and extension to lower sperm concentrations, especially at 25 × 10^6^ sperm/mL, perform poorly under the same cooling conditions. Similar to our results in cooled semen samples, higher sperm concentrations of 200 × 10^6^ sperm/mL were superior in terms of *in vitro* sperm quality parameters, i.e., sperm motility, morphology, and PMI based on propidium iodide fluorescent staining and flow cytometry, for cryopreserved semen in the dog (25, 27).

The poor results of the 25 × 10^6^ sperm/mL group highlight that the dilution effect may cause more harm to the canine spermatozoa over time compared with higher concentrations. Despite the increased glucose and energy sources available for spermatozoa at lower concentrations, a decline in plasma membrane integrity occurs. This showcases the potential role that removal and dilution of seminal plasma factors have on membrane integrity during storage (24, 40), as these factors help stabilize membrane phospholipids when spermatozoa enter a more gel-like state during cooled storage (41–43). Our results therefore pinpoint PMI as an important and potentially more sensitive measure to assess sperm quality than motility and morphology alone in the dog.

After cooling, we subjected all the groups to centrifugation and a second extension to mimic the clinical scenario where the total semen volume required reduction before insemination, e.g., for transcervical insemination, or if lower sperm concentrations in a higher volume would prove superior during cooling in this study. The significant decline in PMI (all groups), CASA motility parameters, and morphologic parameters, as they are related to treatment, indicates that this process induces moderate amounts of damage to the cells. This is likely due to the cells becoming more fragile after cooled storage compared with a raw ejaculate. At this time, it is unknown if these parameters would rebound *in vitro* hours after centrifugation, as it was not investigated in this study. However, these parameters would not have been a reliable representation of *in vivo* processes after semen deposition into the female reproductive tract. Group comparisons at T3 again showed C25 with the lowest performance for all sperm parameters, while CON and C200 generally remained superior or similar to the C50 and C100 groups.

Another clinical aspect of this study was to compare semen quality in terms of processing with centrifugation at the originating or receiving facility. The control group (CON) served this purpose as it was only processed after 24 h of storage (T3) and compared with the treatment groups after 24 h of cooled storage (T2). Based on the significantly lower PMI and NM for CON at T3 compared with the C25-C200 groups and the C100 and C200 groups at T2, respectively (Figure 3), centrifuging after 24 h of cooled storage is not recommended. This includes scenarios of the raw ejaculate not being processed initially and requiring processing at the receiving facility. The centrifugation step after cooled shipment could induce more ROS, resulting in more non-viable and morphologically abnormal spermatozoa in a breeding dose. The presence of non-viable sperm cells may affect the fertilizing ability of viable spermatozoa and induce inflammatory conditions such as endometritis in the bitch (13, 44, 45). Interestingly, motility parameters were not different between the CON group at T3 and the C25–C200 groups at T2. We may speculate that a decline in motility would have followed suit with the decline in PMI had the samples been kept for longer periods after the second processing (T3), similar to reports in other species (21, 23, 24). Again, this observation emphasizes the importance of evaluating sperm motility together with other parameters, i.e., plasma membrane integrity and morphology, for a complete spermiogram.

This study used a commercial canine extender (CaniPlus Chill LT, Minitube) that does not utilize animal products for protein sources. Other extender formulations such as egg yolk-based products may contain higher concentrations of phospholipids and may have different results at different concentrations. Canine sperm cells have a higher proportion of phospholipids within their membranes compared with other species (33, 46), and thus, extender choice could contribute to differences in extracellular and intracellular stabilization of the plasma membrane of spermatozoa. Additionally, the buffering capacity of extender formulas may affect sperm concentration recommendations in the semen sample based on composition and protein type (2, 16, 47–49).

A limitation of this study was that the fertilizing ability of the samples was not assessed, and the parameters measured here are estimations for fertilizing ability (4, 28). Attempting *in vitro* or *in vivo* test breedings would help identify if the decline in quality has a biological impact (50, 51). These practices have been used to assess the effect of processing techniques on bovine, porcine, and equine semen samples; however, these applications are not yet optimized for use in dogs (52, 53).

## 5 Conclusion

Based on our findings, our hypothesis is void due to the generally poorer performance of the lower sperm concentration groups over time, which is in contrast with stallions. Therefore, for cooled shipment, canine semen should be processed and extended to higher sperm concentrations such as 200 × 10^6^ sperm/mL immediately after collection or extended 1:3 vol:vol without centrifugation of the raw ejaculate if volume restrictions allow at the receiving end. If processing is warranted upon arrival, this risks further declines in plasma membrane integrity and normal morphology, resulting in lower numbers of viable spermatozoa for a breeding dose. Adjustments for breeding dose volume should be made on the raw ejaculate before cooling. For dogs with lesser quality semen or those that may perform better in a different extender, testing other extender formulas and different concentration ranges may be indicated. Further studies such as fertilization trials are needed to help understand if the *in vitro* semen parameter changes are biologically significant and correlate with pregnancy rate changes, i.e., *in vivo* proof of concept.

## Data availability statement

The raw data supporting the conclusions of this article will be made available by the authors, without undue reservation.

## Ethics statement

The animal studies were approved by the Institutional Animal Care and Use Committee of Virginia Polytechnic Institute and State University. The studies were conducted in accordance with the local legislation and institutional requirements. Written informed consent was obtained from the owners for the participation of their animals in this study.

## Author contributions

NS: Data curation, Investigation, Writing – original draft. SW: Formal analysis, Writing – review & editing. JTC: Funding acquisition, Investigation, Writing – review & editing. OB: Conceptualization, Funding acquisition, Investigation, Writing – review & editing.
